# Source-Detector Geometry Analysis of Reflective PPG by Measurements and Simulations

**DOI:** 10.1109/OJEMB.2025.3546771

**Published:** 2025-02-28

**Authors:** M. Reiser, T. Mueller, A. Breidenassel, O. Amft

**Affiliations:** ^1^ University of Freiburg, Faculty of Engineering 123115 79110 Freiburg Germany; ^2^ HAW LandshutFaculty of Electrical Engineering 38901 84036 Landshut Germany; ^3^ Ams OSRAM 93055 Regensburg Germany; ^4^ Zentrum für Sonnenenergie- und Wasserstoff-Forschung Baden-Württemberg (ZSW) 557390 89081 Ulm Germany; ^5^ HAW LandshutFaculty of Electrical Engineering 38901 84036 Landshut Germany; ^6^ Hahn-Schickard 199773 79110 Freiburg Germany; ^7^ University of Freiburg, Faculty of Engineering 9174 79110 Freiburg Germany

**Keywords:** Sensor geometry, Monte Carlo simulation, perfusion index, PPG, skin phantom

## Abstract

*Goal:* We investigate the effect of source-detector geometry, including distance and angle, on the reflective photoplethysmography (PPG) signal. *Methods:* A porcine skin phantom was used for laboratory measurements and replicated by Monte Carlo simulations. Variations in sensor geometry were analysed. *Results:* Laboratory measurements and Monte Carlo simulations showed agreement for various geometry settings. With decreasing negative sensor angle, the differential path length factor and the average maximum penetration depth increases. *Conclusions:* Our analyses highlight the influence of source-detector geometry on the PPG DC signal. Based on our analysis of penetration depth and optical path length, the geometry effects can be transferred to the PPG AC signal too. MC simulations provide an important tool to optimise PPG performance.

## I. Introduction

The photoplethysmogram (PPG) provides a non-invasive optical method to detect vital signs in the human body, i.e. to estimate cardiovascular function and respiratory rate. PPG is frequently used in wearable health monitoring devices. In the reflective configuration, a light source, typically a light-emitting diode (LED), directs photons into tissue, and a photodiode detects their reflection at a variable source-detector distance. AC level is caused by a change in blood volume due to the heartbeat. The AC/DC ratio is called perfusion index (PI) and is typically below 10% [1]. DC or signal level, is caused by skin layers, venous blood, and non-pulsatile arterial blood. The DC level is subject to fluctuations caused by changes in capillary density and venous volume fluctuations [2]. The DC level is an important factor in assessing the quality of PPG signals and serves as relative, non-quantitative index of skin vascularity [2], [3]. DC level has a direct impact on PI and can be an indicator for the differential pathlength factor (DPF) as well as penetration depth. Detected photons travel through tissue in a banana-shaped curve between source and detector [4]. The shape, size, and depth of the banana curve is influenced by the source-detector distance $d$[5]. Depending on the penetration depth, several tissue layers with different blood volume fractions are traversed by photons.

Monte Carlo (MC) simulations of photon-tissue interactions are already used to simulate the PPG curve [6]. MC simulations are frequently used to extend the basic understanding of PPG signals [7], [8]. To deal with skin structure complexity, basic layers or voxel models are frequently deployed [9]. Optical skin properties were often modelled from literature data. However, the effects of the optical arrangement between light source and photon detector are still insufficiently understood and comparisons between simulations and measurements are lacking. Moreover, based on simulations, the influence of sensor geometries on optical performance, e.g. on the PI, could be investigated before device development.

In this paper, we aim to increase the fundamental understanding of photon path characteristics and analyse the impact of sensor geometry on DC signal level for wearable devices. Beyond replicating laboratory measurements with MC simulations, we explore the effect of negative source and detector angles, which may improve penetration depth for small geometries. Wearables are subject to strict space restrictions and source-detector distances of far less than 1 cm are therefore adequate. We specifically focus on the DC part of the PPG signal to reduce complexity and to exclude artefacts that may occur due to dynamic effects. In particular, we provide the following contributions:

1) We present laboratory measurements of reflective PPG on a homogeneous porcine skin phantom covering the geometry parameter space spanned by source and detector arrangement (distance and angle) at three frequently used wavelengths: green (520 nm), red (637 nm), and infrared (IR) (940 nm).
2) We detail a voxel-based MC simulation approach, designed to replicate the skin phantom measurements and explore further geometries. We confirm the agreement between simulations and measurements.
3) We analyse penetration depth, optical path length, and differential pathlength factor (DPF) across various geometries (distances and angles), including negative angles of source and detector.

## II. Materials and Methods

To investigate the effects of source-detector geometry, including distance and angle, a previously characterised porcine skin phantom [10] was employed. The porcine skin phantom approximates the absorption and scattering behaviour of real porcine skin [11], [12], [13]. To maintain constant ambient conditions, measurements were performed using an optical laboratory setup. The measurement setup was rebuild using a voxel model and the photon interactions were simulated using a MC simulation framework [14].

### A. Laboratory Setup and Measurements

The cylindrical phantom had a polished surface with a height of 50 mm and a radius of 25 mm. The phantom was optically characterised before, including absorption coefficient $\mu _{a}$, reduced scattering coefficient $\mu
              _{s}\,^{\prime }$, anisotropy factor $g$, and refractive index $n$[11], [12]. Optical parameters are detailed in Table 1.

**TABLE I:** As Absorbing Media, We Considered Glass and the Porcine Skin Phantom

Optical parameters of the measurement setup depending on wavelength $\lambda$.					
Tissue	$\lambda$ [mm]	$\mu _{a} \ [\mathrm{mm^{-1}}]$	$\mu _{s}^{\prime } \ [\mathrm{mm^{-1}}]$	n [-]	g [-]
	520	0.004			
Glass slide	637	0.005	-	1.520	-
	940	0.029			
Air gap	-	-	-	1.000	-
	520	0.093	1.719	1.561	
Phantom	637	0.019	1.376	1.552	0.550
	940	0.016	0.793	1.543	

The Porcine Skin Phantom Was Considered to Be the Only Scattering Medium.

**Fig. 1.**
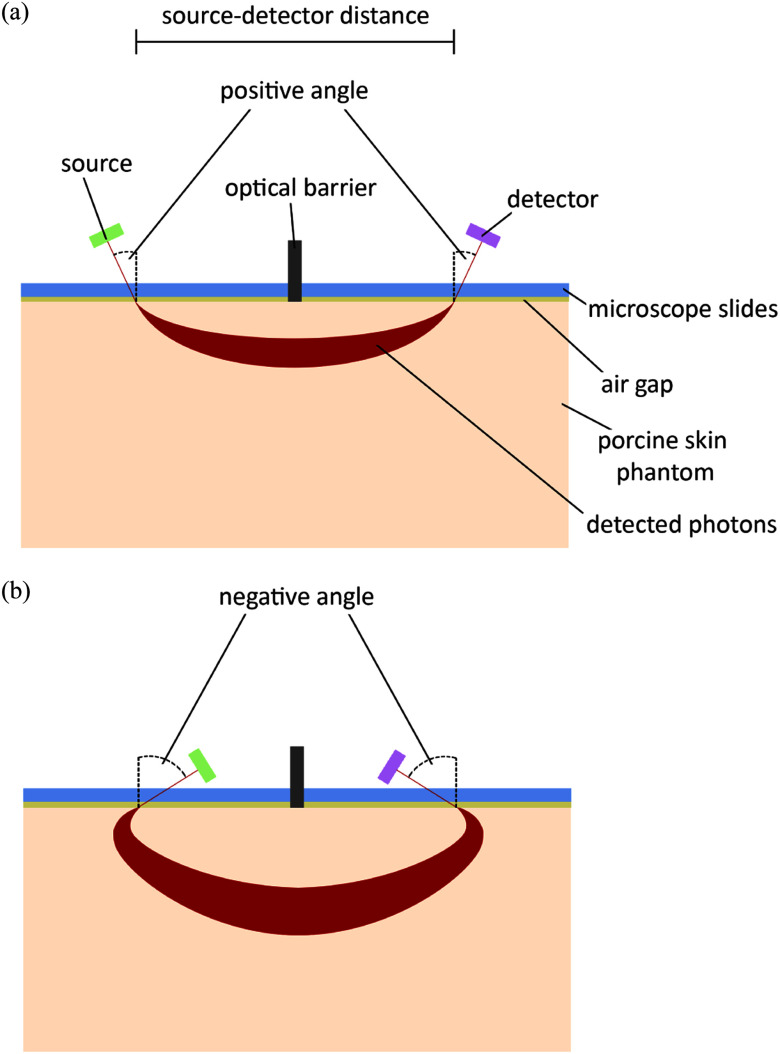
Schematic illustrations of the measurement and simulation setups. (a) Cross-section of the laboratory measurement setup. A cylindrical phantom was used to analyse photon reflection. Microscope slides were placed on the phantom, separated by a vertically arranged optical barrier. Light source angle $\theta _{s}$ was varied between 15$^\circ$ and 55$^\circ$ and detector angle $\theta
                _{d}$ between 25$^\circ$ and 55$^\circ$, both in 10$^\circ$ steps. Source-detector distance d was varied between 2 mm and 5 mm in 1 mm steps. (b) Cross-section of negative source and detector angle setup for MC simulations. Light source angle $\theta _{s}$ was varied between $-15$$^\circ$ and $-55$$^\circ$ and detector angle $\theta
                _{d}$ between $-25$$^\circ$ and $-55$$^\circ$, both in $-10$$^\circ$ steps.

A mechanical frame was used to adjust and maintain positions and orientation of source and detector. Wavelength $\lambda$ was investigated as 520 nm (green), 637 nm (red), and 940 nm (IR). The source angle $\theta
              _{s}$ was increased from 15$^\circ$ to 55$^\circ$ in 10$^\circ$ increments. The facet of the collimated emitter had a diameter of 200 $\mathrm{\mu m}$, which was imaged on the phantom with a magnification factor of $V_{source} =
              3.05$. The detector angle $\theta _{d}$ was increased from 25$^\circ$ to 55$^\circ$ in 10$^\circ$ increments. The detector spot on the phantom had a diameter of 550 $\mathrm{\mu
            m}$ and the photons were collected by a lens with a diameter of 10.9 mm. The detector fibre had a diameter of 200 $\mathrm{\mu m}$ and a magnification factor $V_{detector} =
            2.76$. The source and detector were positioned centrally at the midpoint of the phantom. The source-detector distance was defined as the distance between the source and detector spots projected onto the phantom. The source and detector spots were used to maintain a constant source-detector distance while adjusting the source and detector angles. Angle adjustments of source and detector did not alter the designated source-detector distance, nor did distance adjustments influence source or detector angles. The source-detector distance was adjusted between 2 mm and 5 mm in 1 mm steps. Fig. 1(a) illustrates the laboratory measurement setup.

The porcine phantom was placed centrally under source and detector. An optical barrier was placed vertically onto the phantom and centred between source and detector spot to prevent direct cross-talk. Two microscope slides were positioned on either side of the optical barrier. A small air gap between the microscope slides and the phantom could not be excluded. The microscope slides had a height of 1 mm and covered the phantom except at the optical barrier. The optical barrier had a thickness of 0.5 mm and the outer edge was centred over the phantom. The microstructured phantom was made from epoxy resin [10] and designed as a homogeneous structure to simulate the optical parameters of porcine skin.

### B. Monte Carlo Simulations

Photon-tissue interactions were simulated using a MC algorithm. The porcine skin phantom was implemented as a layered model to reduce computational complexity. The microscope slides, optical barrier, and air gap were implemented in a voxel model with a volume of 0.01 $\text{mm}^{3}$ per voxel. Model dimensions were derived from the laboratory setup. For more details on the MC simulation framework see the supplementary materials. In scattering tissue, the optical path length $l$ differs from the source-detector distance $d$. The relationship is quantified by the differential pathlength factor DPF:

\begin{equation*} l = d \times \text{DPF}. \tag{1} \end{equation*}

For non-scattering tissue, the Lambert-Beer law of absorption is applied for $d$ and $\text{DPF} =
            1$ as well as $l
              = d$.

### C. Experiments

Laboratory measurements and MC simulations of positive angles were repeated five times for each parameter setting (i.e. wavelength $\lambda$, source-detector distance $d$, source angle $\theta
          _{s}$, and detector angle $\theta _{d}$). Measurements were performed for source angles from 15$^\circ$ to 55$^\circ$ in 10$^\circ$ steps and detector angles 25$^\circ$ to 55$^\circ$ in 10$^\circ$ steps. MC simulations were performed and validated accordingly, including 0$^\circ$ for source and detector angles. Negative source and detector angles were analysed using MC simulations (see Fig. 1(b)). For negative angles, the detector angle $\theta
            _{d}$ was decreased from $-25$$^\circ$ to $-55$$^\circ$ and the source angle $\theta _{s}$ from $-15$$^\circ$ to $-55$$^\circ$, both in $-10$$^\circ$ increments.

Simulations were performed separately for HG and Mie (see supplementary materials) [15]. The particle size of TiO$_{2}$ scatterers of the phantom was 250 nm and the refractive indices of the scatterers were derived according to DeVore [16].

Simulated metrics for photon packets included detected weight $w$ at the detector surface, maximum penetration depth, and optical path length $l$. In the laboratory setup, DC signal level at the detector was measured, which supposedly corresponds to the detected weight $w$ of simulated photon packets.

The number of photon packets had to be increased linearly with source-detector distance $d$ to obtain sufficient signal. With increased source-detector distance $d$ and negative source and detector angles, the shot noise to detected photon packets increased too, which was smoothed by multiple simulation seeds. Relative DC signal level was unaffected by the increase in photon packets, since we applied a normalisation depending on the source-detector distance. With the normalisation, laboratory measurements and MC simulations can be directly compared for a given source and detector angle configuration (see Table 2).

**TABLE II:** Source and Detector Angle Configurations Used to Derive the DC Signal Normalisation Level $\bar{S}_{\text{norm},
                  \lambda, d}$ (see (2))

Sensor angle configuration for DC signal level normalisation		
Detector surface plot type	Source angle $\theta _{s}$	Detector angle $\theta _{d}$
Positives angles	15$^\circ$	25$^\circ$
Negative angles	-55$^\circ$	-55$^\circ$

Measured and simulated DC signal levels for positive angles were normalised by the DC signal level at a source angle of 15$^\circ$ and detector angle of 25$^\circ$. DC signal levels for simulated negative angles were normalised at a source angle of $-15$$^\circ$ and detector angle of $-25$$^\circ$ of the respective parameter configuration (i.e. source-detector distance $d$ and wavelength $\lambda$). To compare laboratory measurement and MC simulations, we used:

\begin{equation*} \bar{S}^{\prime }_{\lambda, d, \theta _{s}, \theta
              _{d}} = \frac{\bar{S}_{\lambda, d, \theta _{s}, \theta _{d}}}{\bar{S}_{\text{norm},
              \lambda, d}}, \tag{2} \end{equation*}

where $\bar{S}_{\lambda, d, \theta _{s}, \theta
              _{d}}$ is the average DC level for wavelength $\lambda$, source angle $\theta
            _{s}$, detector angle $\theta _{d}$, and source-detector distance $d$. $\bar{S}_{\text{norm}, \lambda,
              d}$ is the DC signal level at a given source and detector angle (see Table 2).

## III. Results

**Fig. 2.**
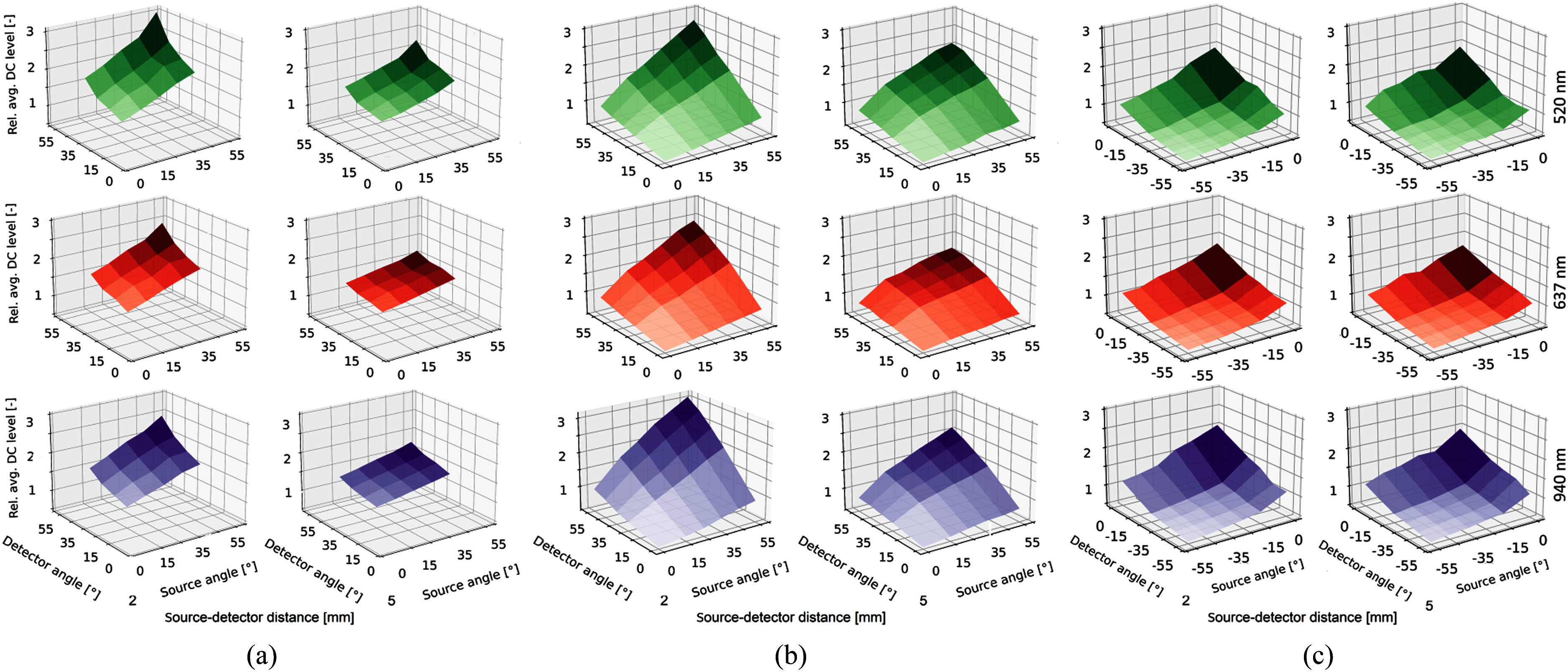
Exemplary comparison of average DC level between laboratory measurements and MC simulation of HG for all wavelengths, measured angles, and source-detector distances $d={\text{2
              mm}}$ and $d={\text{5 mm}}$. DC level was measured and simulated at the detector surface. Positive angle plots were normalised to the respective DC level at source angle of 15$^\circ$ and detector angle of 25$^\circ$. Negative angle plots where normalised at a source angle of $-15$$^\circ$ and detector angle of $-25$$^\circ$. (a) Laboratory measurements. (b) MC simulations of HG for positive angles. (c) MC simulations of HG for negative angles.

The relative DC levels (e.g., of the detector surface plots shown in Fig. 2) can be compared with each other due to their normalisation. Detector surface plots show the illumination fraction at the given source and detector angle. Fig. 2(a), (b) and (c) show that DC signal levels of source and detector angles for both laboratory measurements and MC simulations increased linearly with increasing source and detector angles for all wavelengths. The source had a more prominent influence on the signal increase for increasing angle compared to the detector. For source and detector angle at 55$^\circ$ an outlier can be observed for the laboratory measurements. The slope of the signal increase intensified as the source and detector angles increased, but diminished as the source-detector distance $d$ increased. The relative maximum signal, which was both measured and simulated at a source and detector angle of 55$^\circ$ for each source-detector distance $d$, decreased with increasing source-detector distance $d$. Measurements and simulations of HG were in agreement for 520 and 637 nm. Deviations between laboratory measurements and simulations of HG were found at 940 nm. For positive angles, the detected DC signal level decreased with increasing source-detector distance $d$, by an average factor of 0.04 for 520 nm, 0.09 for 637 nm, and 0.15 for 940 nm (see Fig. 2 in supplementary materials).

The laboratory setup required precise positioning of the source and detector to maintain a constant source-detector distance $d$ for repeated measurements. Deviations in positioning of source and detector due to the setup resulted in variations of measured DC level, e.g. see deviations of the linear DC level increase in Fig. 2(a) at 520 nm and 2 mm.

Fig. 2(c) shows that DC signal level also increases with increasing negative source and detector angles.

**Fig. 3.**
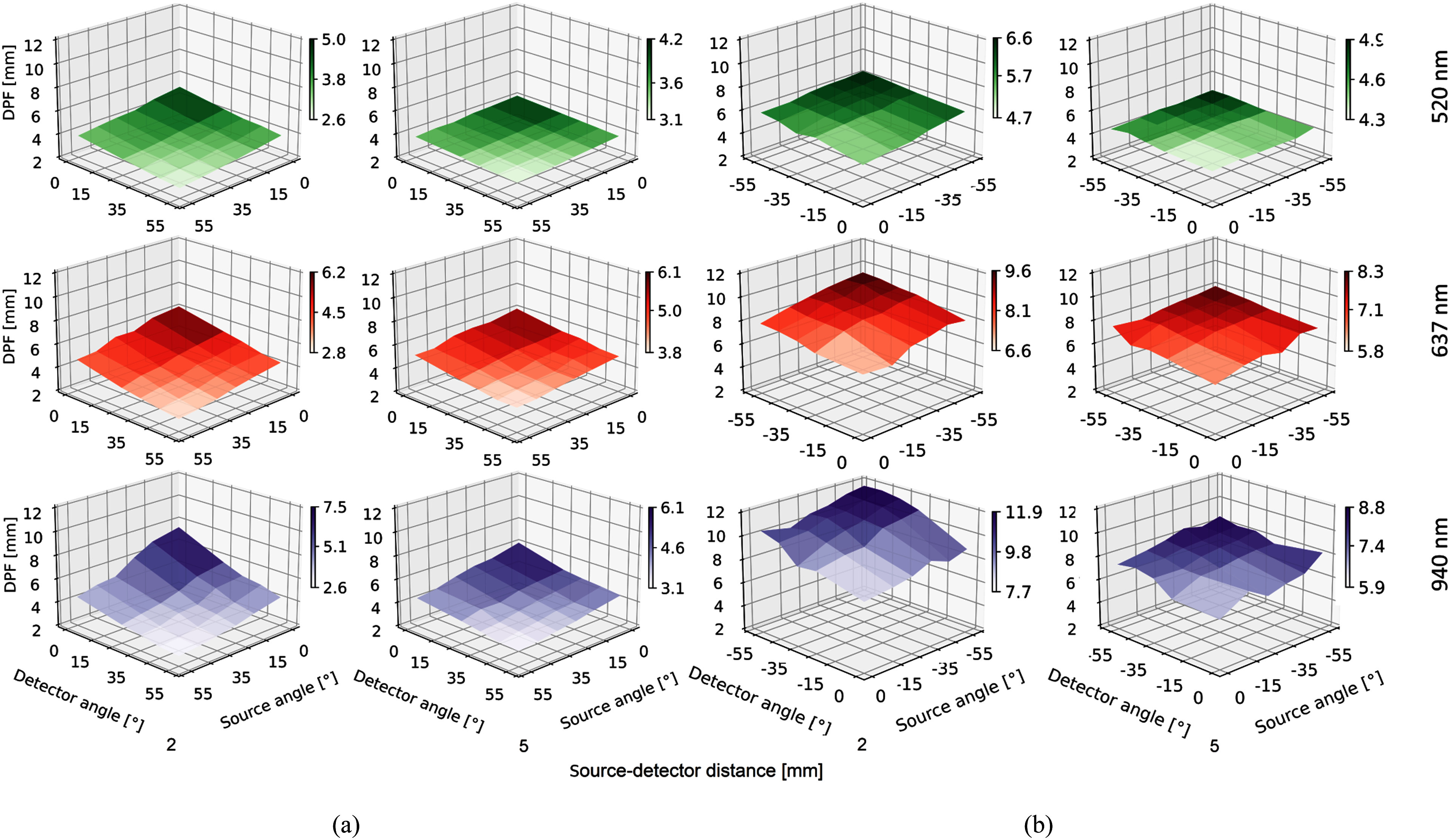
Average differential pathlength factor (DPF) for positive and negative source and detector angles at source detector distance $d={\text{2 mm}}$ and $d={\text{5 mm}}$. Diagrams show results of MC simulation of HG plotted for all wavelength. (a) Positive angles. (b) Negative angles.

In Fig. 3 we analysed positive and negative source and detector angles by exploratory MC simulation. Overall, DPF increased for source angles $\theta _{s}$ from 55$^\circ$ to 0$^\circ$ and 0$^\circ$ to $-55$$^\circ$ and detector angles $\theta _{d}$ from 55$^\circ$ to 0$^\circ$ and 0$^\circ$ to $-55$$^\circ$ and rising wavelength, suggesting that penetration depth can be tuned, e.g. for small form factor sensor designs. DPF decreased with increasing source-detector distance $d$. The source angle $\theta _{s}$ had a more prominent effect on DPF than the detector angle $\theta
            _{d}$ for all wavelengths. In general, 940 nm yielded the highest DPF, followed by 637 nm and 520 nm.

**Fig. 4.**
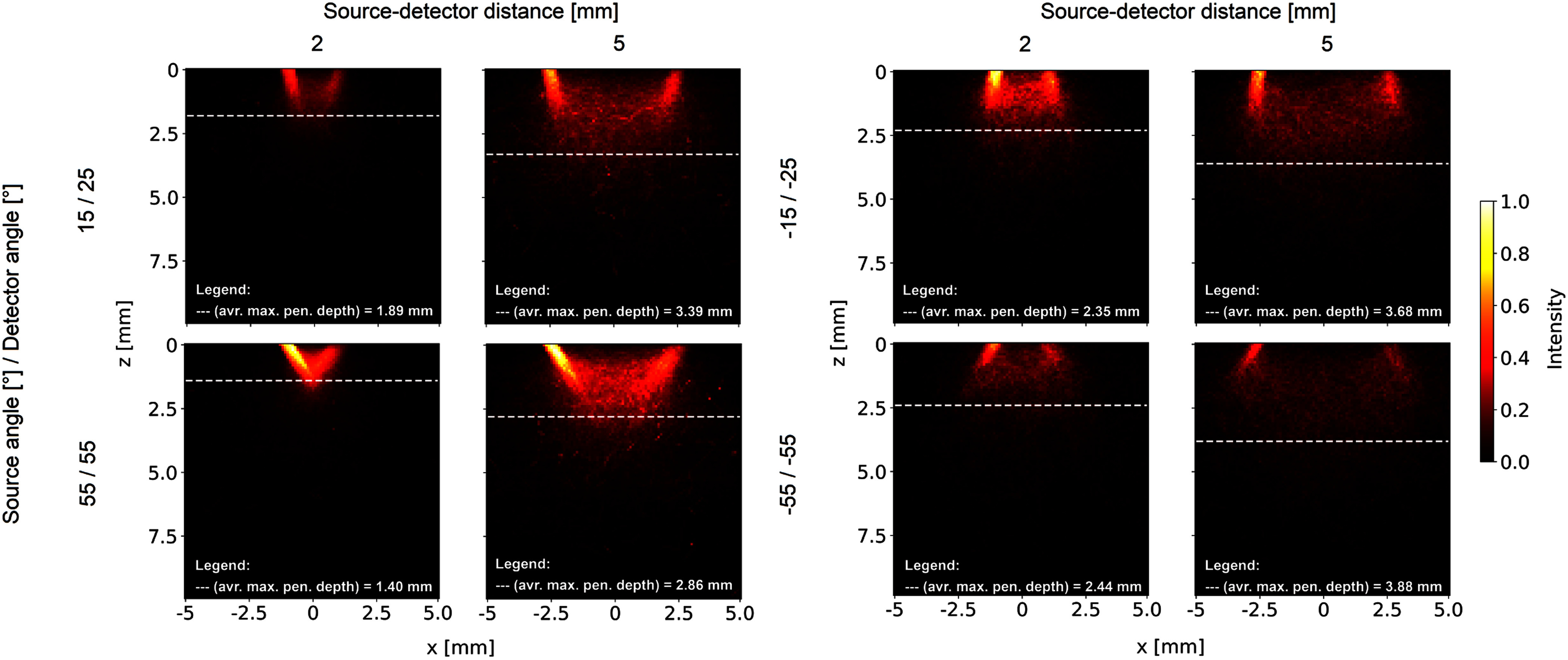
Example photon plots of the source-detector distance, source angle, and detector angle at 520 nm. Source-detector distances were 2 mm and 5 mm. Source angles were 55$^\circ$, 15$^\circ$, $-15$$^\circ$, and $-55$$^\circ$. Detector angles were 55$^\circ$, 25$^\circ$, $-25$$^\circ$, and $-55$$^\circ$. The intensity of the plots has been normalised to the maximum value of the visualised data to illustrate differences in detected photons traversing through the phantom.

Fig. 4 shows photon plots within $-5$ to $+5$ mm from the centre of the phantom for parameter limits, 2 and 5 mm source-detector distance $d$. Photon plots were normalised to the maximum value at a source-detector distance $d$ of 2 mm, source angle $\theta _{s}$ of 15$^\circ$ and detector angle $\theta
          _{d}$ of 55$^\circ$ for positive angles, source angle $\theta _{s}$ of $-15$$^\circ$, and detection angle $\theta _{d}$ of $-25$$^\circ$ for negative angles. Penetration depth increased with rising source-detector distance $d$ for negative and positive angles. Penetration depth decreased with increasing positive source and detector angles and increased with decreasing negative source and detector angles.

## IV. Discussion

The present study analysed the effect of source-detector geometry on the DC signal of reflective PPG and compared our theoretical understanding represented by MC simulations to measurements at a skin phantom.

The DC signal level was influenced by source and detector angle. With increasing source and detector angles, the DC signal level increased. In a reflective PPG system, detected photons travel through tissue in a banana curve [17]. The banana curve depth was influenced by the source angle $\theta _{s}$. Penetration depth decreased with increasing source and detector angles, i.e. from 15$^\circ$ to 55$^\circ$ and from 25$^\circ$ to 55$^\circ$, and increased with rising source-detector distance $d$, which was consistent with observations of Chatterjee et al. [18]. Thus, for increased positive angles, detected photon packets travelled with a shorter path through tissue and experienced a reduced absorption. With increased detector angles $\theta _{d}$, the skin surface area of the reflected beam increased and thus more photon packets could be detected. DC level variation was larger for source angle changes than for a change of detector angles. With increasing source-detector distance $d$, the DC level decreased but the penetration depth increased. The observed decrease in penetration depth resulting from an increased detector angle $\theta _{d}$, i.e. $\theta _{d}$ towards 55$^\circ$, demonstrates that the gain in DC level is not solely attributed to an expansion in detector space. The morphology of the phantom had no influence on the shape of the banana curve, as the photon packets heading toward the edge of the cylindric phantom did not reach the detector; photons were absorbed on the path to the detector or left the phantom without being detected.

If the space between source and detector is minimal, the typical banana curve gets very flat, i.e. penetration depth is reduced, compared to wider source-detector distances. Thus, we hypothesised that at source-detector distances of, e.g., $2-3$ mm, negative angles may increase penetration depth. Our exploration results show that DC signal level decreases for negative source angles from $-15$$^\circ$ to $-55$$^\circ$ and detector angles from $-25$$^\circ$ to $-55$$^\circ$, eventually reach a minimum at a source angle $\theta
          _{s}$ of $-55$$^\circ$ and a detector angle $\theta _{d}$ of $-55$$^\circ$ for all source-detector distances $d$ and wavelengths. However, for negative angles, the smaller the source and detector angles, the larger are penetration depth and optical path length $l$ through tissue, thus may reach a larger fraction of blood volume.

Overall, we showed that pointing source and detector towards each other increases DC signal level, but decreases penetration depth, DPF, and optical path length $l$. Conversely, negative angles, where source and detector point in opposite directions (i.e. away from each other), lower the DC signal level, but increase the penetration depth, DPF, and optical path length $l$. We hypothesise that due to the negative angles, photons reach deeper layers. Thus, the shape of the banana curve can be influenced by changing the sensor geometry.

Human skin consists of several layers with different blood volume fractions [19]. Deeper layers, including upper and deep blood net dermis, carry a larger blood volume than more superficial skin layers. Our results indicate that specific source-detector distances $d$ as well as source and detector angles can increase penetration depth of the PPG curve. Photons reach deeper skin layers that carry higher blood volume with increasing source-detector distance $d$ and decreasing angles. When penetration depth rises, the signal quality (e.g. DPF and PI), can be improved in reflective PPG measurements.

In this work, we investigated the influence of sensor geometry on the PPG curve. In particular, we focused on the PPG signal's DC component to minimise the parameter space. Since increased penetration depth results in more photons that interact with blood, the quality of the PPG signal's AC component will increase too. Thus our results warrant further investigation on the AC component, in particular to analyse the effect of key parameters, including skin layer structure, melanin content, BMI, blood glucose, dynamic blood volume, etc. Given the performance of our simulations, further AC component investigations should be based on MC simulations too, but will require additional validation analyses.

Several skin models were used for MC simulations, which differ in layers (3-layer, 6-layer, 7-layer, etc.), absorption and scattering coefficients, anisotropy factors, and refractive indices [8], [20]. Detailed skin models and their uncertainties should be addressed in future studies. In this work, we focused on fundamental optical aspects of skin and demonstrated that real-world measurements can be explained by MC simulations for a porcine skin phantom.

PPG measurement devices require calibration for their specific sensor arrangement. Calibration is a common requirement of PPG devices that measure oxygen saturation. To adjust for changes in DPF and optical path length, calibration would be needed for a device that used negative sensor angles too.

In this paper, we compared positive source and detector angles (15$^\circ$ to 55$^\circ$) in laboratory measurements and MC simulations. The Lambertian emission profile of an LED and the full field of view of a conventional photodiode affect the DC signal level. When we simulated negative angles, only source and detector angles were changed while the model setup remained the same. Given the agreement of measurements and simulations for the wide range of positive angles in this work, we did not find reasons to assume that our simulation approach would not transfer to negative angles. Our exploratory results suggest that future research may investigate negative angle effects in measurements. Another advantage of our MC simulations is that it is insensitive to measurement variations due to individual anatomy.

## V. Conclusion

In this paper, we demonstrated an agreement between laboratory measurements of a porcine-skin phantom and MC simulations for light at 520 nm, 637 nm, and 940 nm. We conclude that MC simulations offer the potential for device design exploration. We analysed quantities that cannot be derived from measurements, including DPF and penetration depth. To investigate PPG sensor performance, subsequent work may consider further tissue models.

We validated MC simulations with measurements and then demonstrated that not only the source-detector distance influences the reflective PPG signal banana curve, but the source and detector angle too. Negative source and detector angles may increase DPF and penetration depth of detected photons. The photon path of negative angled source and detector could reach a larger fraction of blood volume and could increase the PI too. As a result, negative source and detector angles could improve the PPG signal, if other geometric optimisations, e.g. increase of source-detector distance, is not an option due to size limits of device or measurement site.

We conclude that realistic photon-tissue interactions can be simulated. Simulations can used, e.g., to optimise PI with respect to sensor geometry. The best choice of phase function in simulations depends on tissue, scatterer characteristics (e.g. scattering coefficient $\mu _{s}^{\prime
          }$), and angular resolution. In our setup, HG yielded the highest agreement between laboratory measurements and MC simulations.

## Supplementary Materials

We summarise optical parameters. A comprehensive examination of the agreement between laboratory measurements and MC simulation is provided. DC signal level from laboratory measurements, the decrease of the DC signal level with increasing source-detector distance, as well as the effect of phase functions (Henyey-Greenstein, Mie theory) on simulation results were investigated.

Epoxy-resin based optical phantom absorption may increase with age [10]. We analyse non-aged and aged phantoms with the HG phase function.

We analyse the signal-to-noise ratio (SNR) for different sensor configurations by evaluating the impact of source and detector angles (55$^\circ$, 0$^\circ$, $-55$$^\circ$) and source-detector distances (2 mm to 5 mm). The required optical input power was examined for sensor configurations of $-55$$^\circ$ and 0$^\circ$ to match the SNR of 55$^\circ$, while ensuring compliance with the maximum permissible exposure (MPE) according to IEC 60825-1. Figures and Tables are presented for MC simulations of positive and negative source and detector angles for source-detector distances $d={\text{3
            mm}}$ and $d={\text{4 mm}}$ and for all wavelengths.

Supplementary Materials

## Author Contributions

MR developed the Monte Carlo simulation algorithm, conducted the simulations, supported TM with laboratory measurements, analysed and interpreted both laboratory and simulation results, and drafted as well as revised the manuscript. TM carried out the laboratory measurements, interpreted the measurements, and contributed to the manuscript review. AB and OA provided interpretation of both laboratory and simulation results and supported editing and reviewing the manuscript.

## Conflict of Interest

There are no conflicts of interest to declare.
