# Supplementary material for: Source-Detector Geometry Analysis of Reflective PPG by Measurements and Simulations
Source: IEEE Open J Eng Med Biol. 2025 Feb 28;6:400–6. doi: 10.1109/OJEMB.2025.3546771 (PMC12251171; doi:10.1109/OJEMB.2025.3546771)
Supplement: Supplementary Materials [file supp1-3546771.pdf]

Supplementary Materials

Source-detector geometry analysis of reflective PPG by measurements and simulations

M. Reiser*, T. Mueller, A. Breidenassel, and O. Amft

THE Supplementary Materials provide detailed optical parameters. A comprehensive examination of the agreement between laboratory measurements and Monte Carlo simulations is provided. The deviations of DC signal level in the laboratory, the decrease of the DC signal level due to the increasing source-detector distance, as well as the effect of the phase functions (Henyey-Greenstein and Mie theory) on the simulations were investigated.

Figures and Tables are presented for Monte Carlo simulations of negative and positive source and detector angles for source-detector distances d from 2 to 5 mm and all wavelength.

I. Monte Carlo simulations

Reduced scattering coefficient ${\mu_s}'$ [1] represented the probability of scattering events of photons in tissue, including the anisotropy factor g and scattering coefficient μ_s:

$$ {\mu_s}' = \mu_s(1-g). \tag{1}$$

Photons were bundled as photon packets and emitted with a statistical weight $w = 1.0$. In glass and tissue, w was reduced by the absorption coefficient μ_a. First, photon packets were sent to the microscope slides with a parameterised source angle. Path length l was calculated depending on ${\mu_s}'$ of the tissue:

$$ l = -\frac{\xi}{{\mu_s}'}, \tag{2}$$

where ξ was a uniformly distributed random number between 0 and 1. Subsequently, the absorbance was determined and w reduced by Lambert-Beer's law [2]:

$$ w = w' \cdot e^{-\mu_a \cdot \Delta l}, \tag{3}$$

where Δl was the travelled length inside the tissue. After each travelled path length l in scattering tissue, a new scattering angle θ was calculated using two approaches: (1) analytically using the Henyey-Greenstein phase function (HG) [2]:

$$ p(\theta) = \frac{1}{4\pi} \frac{1-g^2}{(1+g^2-2g\ cos\theta)^{\frac{3}{2}}}, \tag{4}$$

where g was the anisotropy factor, and (2) numerically using Mie Theory [3] and a lookup table (Mie) [4]. The azimuth angle ϕ was chosen randomly between 0 and 2π. In tissue without scattering, photon packets were directed to the next tissue boundary and w was reduced according to Eq. 3.

For tissue transitions, reflectance R [2] was calculated based on the incident angle θ_i, transmission angle θ_t, and critical angle θ_c:

$$ R = \begin{cases} \frac{(n_i-n_t)^2}{(n_i+n_t)^2} & \text{if } \theta_i = 0, \\ \frac{1}{2}\left[\frac{sin^2(\theta_i-\theta_t)}{sin^2(\theta_i+\theta_t)} + \frac{tan^2(\theta_i-\theta_t)}{tan^2(\theta_i+\theta_t)^2}\right] & \text{if } 0 < \theta_i < \theta_c, \\ 1 & \text{if } \theta_c < \theta_i < \frac{\pi}{2}, \end{cases} \tag{5}$$

where n is the refractive index (i: incident, t: transmitted). If R was smaller than a random number ξ, the photon packet was transmitted into the next tissue, otherwise reflected.

Each photon packet propagated through the tissue until it left the model's virtual boundaries (i.e., the skin phantom or the microscope slides) or its weight w decreased to a low photon weight threshold $w < 10^{-4}$. If photon packets did not hit the detector surface area after exiting the microscope slide at the specific detector angle, they were discarded. If w was below the low photon weight threshold, the Russian roulette technique [2] was applied to simulate the energy conservation principle. Photon packets had a chance of 1:10 to tenfold their weight w. Photon packets were processed in iteration until the photon packet count of 10^9 was reached.

II. Phantom ageing

Epoxy-resin based optical phantom absorption may increase with age [5]. To represent phantom ageing of our porcine skin phantom, we assumed an age of one year and increased the absorption coefficient μ_a for wavelengths 520 nm and 637 nm according to Krauter et al. [5]. The ageing effect of the phantom at 940 nm was neglectable. The refractive index n, anisotropy factor g, and scattering coefficient μ_s are not affected by ageing.

III. Validation of Monte Carlo simulations

We determined the area under the curve (AUC) from the normalised surface plots to provide a more comprehensive assessment of the congruence between laboratory measurements and MC simulation. AUC represented the integral of the normalised DC signal level between the source angle θ_s of 15° to 55° and the detector angle θ_d of 25° to 55° for source-detector distances d of 2 to 5 mm. As the source-detector distance d increased, the calculated AUC showed a linear decrease across all wavelengths (see Fig. 1). The best agreement between laboratory measurements and MC simulations was achieved for simulations with HG at 520 nm and 637 nm, with a deviation of 1.1% ± 1.1% and 2.6% ± 2.0%, respectively. Furthermore, Mie at 940 nm showed deviations of 8.3% ± 2.5% (see Tab. I). Across all wavelengths, HG simulations reproduced the measurement results most accurately with an average deviation of 7.0% ± 1.2%.

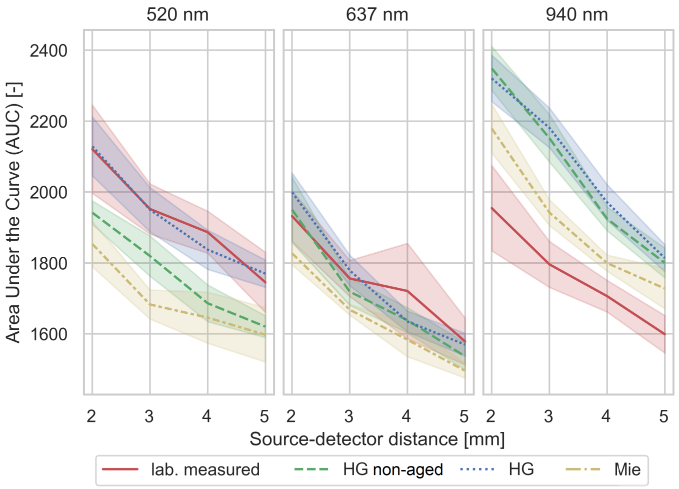

Fig. 1. Area under the curve (AUC) of detector surface plots (Fig. 4) for comparison of laboratory measurements and MC simulation, with source-detector distances d between 2 mm to 5 mm.

AVERAGE PERCENTAGE DEVIATION AND STANDARD DEVIATION OF AUC PLOTS

Wavelength [mm]	HG non-aged $\mu \pm$ STD [%]	HG $\mu \pm$ STD [%]	Mie $\mu \pm$ STD [%]
520	8.3 ± 1.7	1.1 ± 1.1	11.9 ± 2.4
637	2.6 ± 1.6	2.6 ± 2.0	5.9 ± 1.4
940	16.3 ± 4.2	17.3 ± 3.5	8.3 ± 2.5
Total	9.1 ± 1.5	7.0 ± 1.2	8.7 ± 0.6

TABLE I. Average percentage deviation and standard deviation of MC Simulations against laboratory measurements of AUC plots across source-detector distance d for all wavelengths.

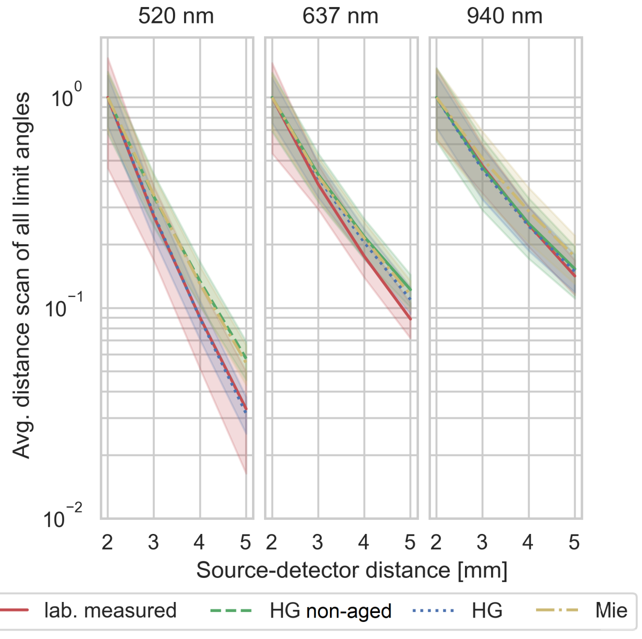

Fig. 2. Measured and simulated relative averaged DC signal levels across all angle configurations, source-detector distance d, and wavelength.

AVERAGE PERCENTAGE DEVIATION AND STANDARD DEVIATION OF DISTANCE PLOTS

Wavelength [mm]	HG non-aged $\mu \pm$ STD [%]	HG $\mu \pm$ STD [%]	Mie $\mu \pm$ STD [%]
520	48.7 ± 20.2	1.3 ± 2.8	45.2 ± 16.0
637	24.3 ± 10.8	15.8 ± 6.7	23.5 ± 8.7
940	4.6 ± 4.3	1.8 ± 4.0	18.3 ± 6.5
Total	25.9 ± 18.0	6.3 ± 6.7	29.0 ± 11.7

TABLE II. Average percentage deviation and standard deviation of laboratory measurements and MC simulations of distance plots for all wavelengths. Results were normalised to the DC level at 2 mm. Top: Deviation of MC simulations against laboratory measurements. Bottom: Average error across source-detector distance d.

In Fig. 2 signals were normalised to their maximum value at 2 mm for each wavelength and the average of all angle configurations per wavelength and source-detector distance d is shown. The relative average DC level decreased with increasing source-detector distance d independent of wavelength and source and detector angle configuration. The slope of the decrease in DC level decreased with increasing wavelength. The average percentage deviation of MC simulations and laboratory measurements could be reduced from $25.9 \pm 18.0\%$ to $6.3 \pm 6.7\%$ by accounting for epoxy resin ageing (see Fig. 2).

Tab. II shows the average DC signal level deviation between MC simulations and laboratory measurements across source-detector distance d and wavelength (see Fig. 2). The best match between MC simulations and laboratory measurements was achieved for HG across all wavelengths, with a deviation of $1.3\% \pm 2.8\%$ for 520 nm, $15.8\% \pm 6.7\%$ for 637 nm, and $1.8\% \pm 4.0\%$ for 940 nm. Mie simulations performed worst with an overall deviation of $29.0\% \pm 11.7\%$, compared to "HG non-aged" and HG with $25.9\% \pm 18.0\%$ and $6.3\% \pm 6.7\%$, respectively.

MC simulations with phase functions according to Henyey-Greenstein and Mie theory were analysed. Profound differences were observed between HG and Mie scattering models within the sub-diffuse backscattering regime, even when considering the same anisotropy factor g [6]. Consequently, relying solely on g as an independent quantifier for sub-diffusive behaviour was considered inadequate [7]. According to Calabro and Bigio [8], the signal is composed of diffuse and sub-diffuse regimes in a source-detector distance range between 2 to 5 mm. For small source-detector distances d, the photon packets travelled in the sub-diffuse regime primarily and thus were not sufficiently scattered. Thus, the 1st order moment of the Legendre polynomial, which is equivalent to g, was no longer sufficient to describe the scattering [8], [9]. HG underestimated backscatter due to high angles [6], [9]. A phase function that also takes into account Rayleigh scattering can be used to additionally consider high-angle scattering [8]. Mie was of limited applicability in our investigation, since phantom scatterer particles were not spherical, but polygonal and rectangular [5]. Furthermore, the scatterer showed anisotropy in form of birefringence. Compared to the other wavelengths, simulations at 940 nm showed a higher angle-dependent signal increase than laboratory measurements. Canpolat et al. [9] showed that scattering coefficient $\mu_s{}'$ is insufficient to describe photon-tissue interactions if the product of the source-detector distance d and $\mu_s{}'$ is less than 2, which applied to a wavelength of 940 nm. Thus, deviations between laboratory measurements and MC simulations, as observed in our case, are to be expected.

IV. Signal-to-Noise Ratio

We examined the signal-to-noise ratio (SNR) of the DC signal level for different sensor configurations (e.g., maximum positive angle, perpendicular, and maximum negative angle) to quantify the loss in signal quality. We analysed the increase in optical input power of our setup to elevate the SNR of configurations with source and detector angles of $-55°$ and $0°$ to match the SNR level at $55°$. We compared the required optical peak power and average input power up to the maximum permissible exposure (MPE) according to IEC 60825-1.

Fig. 3 shows the SNR analysis results. The highest SNR was obtained for source and detector angles of $55°$, followed by $0°$ and $-55°$, across all wavelengths. SNR decreased with a source-detector distance from 2 to 5 mm for all wavelengths (see Fig. 3A). SNR was mostly affected by decrease in source and detector angle as well as the source-detector distance. SNR decrease for source-detector distance was most profound at 520 nm.

Optical input power was below MPE peak and average optical power (see Fig. 3B). Power at source-detector angle of $-55°$ that was needed to match the SNR at $0°$ as well as the SNR at $55°$ was still below MPE limits.

We analysed SNR for three source-detector angles ($-55°$, $0°$, and $55°$), as well as source-detector distance and wavelength. While SNR was lowest for $-55°$, there is a clear correspondence with source-detector distance. For example, SNR at 520 nm, $-55°$, and a source-detector distance of 2 mm, corresponds to the SNR at $0°$ and 3 mm for the same optical power, thus a distance reduction of $\sim$1 mm at $-55°$. The effect is even amplified for 637 nm (distance reduction: $\sim$1.5 mm) and 940 nm (distance reduction: $\sim$3 mm). Further analyses showed that the optical power needed at $-55°$ to match the SNR at $0°$ as well as the SNR at $55°$, stood within the MPE permissible limits.

V. Supplementary results

Fig. 4 shows the DC signal level for positive and negative source and detector angles of the wavelengths 520, 637 and 940 nm. The average of the DC signal level of the passing seeds is shown. The plots are normalised to the respective lowest DC signal level at a source angle of $-55°$ and a detector angle of $-55°$ per source-detector distance d to increase the comparability.

Fig. 5 shows the average maximum penetration depth of HG for positive source and detector angles. The penetration depth decreased with increasing source and detector angle and increases with increasing source-detector distance for all wavelengths.

Fig. 6 shows the average maximum penetration depth of HG for negative source and detector angles. The penetration depth increased for decreasing source and detector angles and source-detector distance for all wavelength.
Fig. 7 shows the DPF of HG for positive source and detector angles. DPF increased with decreasing source and detector angle.

References

[1] T.J. Farrell, M.S. Patterson and B. Wilson (1992, Jul.). A diffusion theory model of spatially resolved, steady-state diffuse reflectance for the noninvasive determination of tissue optical properties in vivo. *Med. Phys.*. vol. 19, no.4, pp. 879-888, doi: 10.1118/1.596777

[2] M. Reiser, A. Breidenassel and O. Amft (2022, Sep.). Simulation framework for reflective PPG signal analysis depending on sensor placement and wavelength. Presented at IEEE-EMBS International Conference on Wearable and Implantable Body Sensor Networks (BSN), doi: 10.1109/BSN56160.2022.9928522

[3] G. Mie (1908). Beiträge zur Optik trüber Medien, speziell kolloidaler Metallösungen *Ann. Phys.*. vol. 330, no. 3, pp. 377-445, doi: 10.1002/andp.19083300302

[4] P. Naglič, F. Pernuš, B. Likar and M. Bürmen (2017, Mar.). Lookup table-based sampling of the phase function for Monte Carlo simulations of light propagation in turbid media. *Biomed. Opt. Express*. vol. 8, no. 3, pp. 1895, doi: 10.1364/BOE.8.001895

[5] P. Krauter, S. Nothelfer, N. Bodenschatz, E. Simon, S. Stocker, F. Foschum and A. Kienle (2015, Oct.). Optical phantoms with adjustable subdiffusive scattering parameters. *Journal of Biomedical Optics*. vol. 20, no. 10, pp. 105008, doi: 10.1117/1. JBO.20.10.105008

[6] J.R. Mourant, J. Boyer, A.H. Hielscher and I.J. Bigio (1996, Apr.). Influence of the scattering phase function on light transport measurements in turbid media performed with small source–detector separations. *Opt. Lett.*. vol. 21, no. 7, pp. 546, doi: 10.1364/OL.21.000546

[7] N. Bodenschatz, P. Krauter, A. Liemert and A. Kienle (2016, Mar.). Quantifying phase function influence in subdiffusively backscattered light. *J. Biomed. Opt.*. vol. 21, no. 3, pp. 035002, doi: 10.1117/1. JBO.21.3.035002

[8] K.W. Calabro and I.J. Bigio (2014, Jul.). Influence of the phase function in generalized diffuse reflectance models: review of current formalisms and novel observations. *J. Biomed. Opt.*. vol. 19, no. 7, pp. 075005, doi: 10.1117/1. JBO.19.7.075005

[9] M. Canpolat and J.R. Mourant (2000, May). High-angle scattering events strongly affect light collection in clinically relevant measurement geometries for light transport through tissue. *Phsy. Med. Biol.*. vol. 45, no. 5, pp. 1127-1140, doi: 10.1088/0031-9155/45/5/304

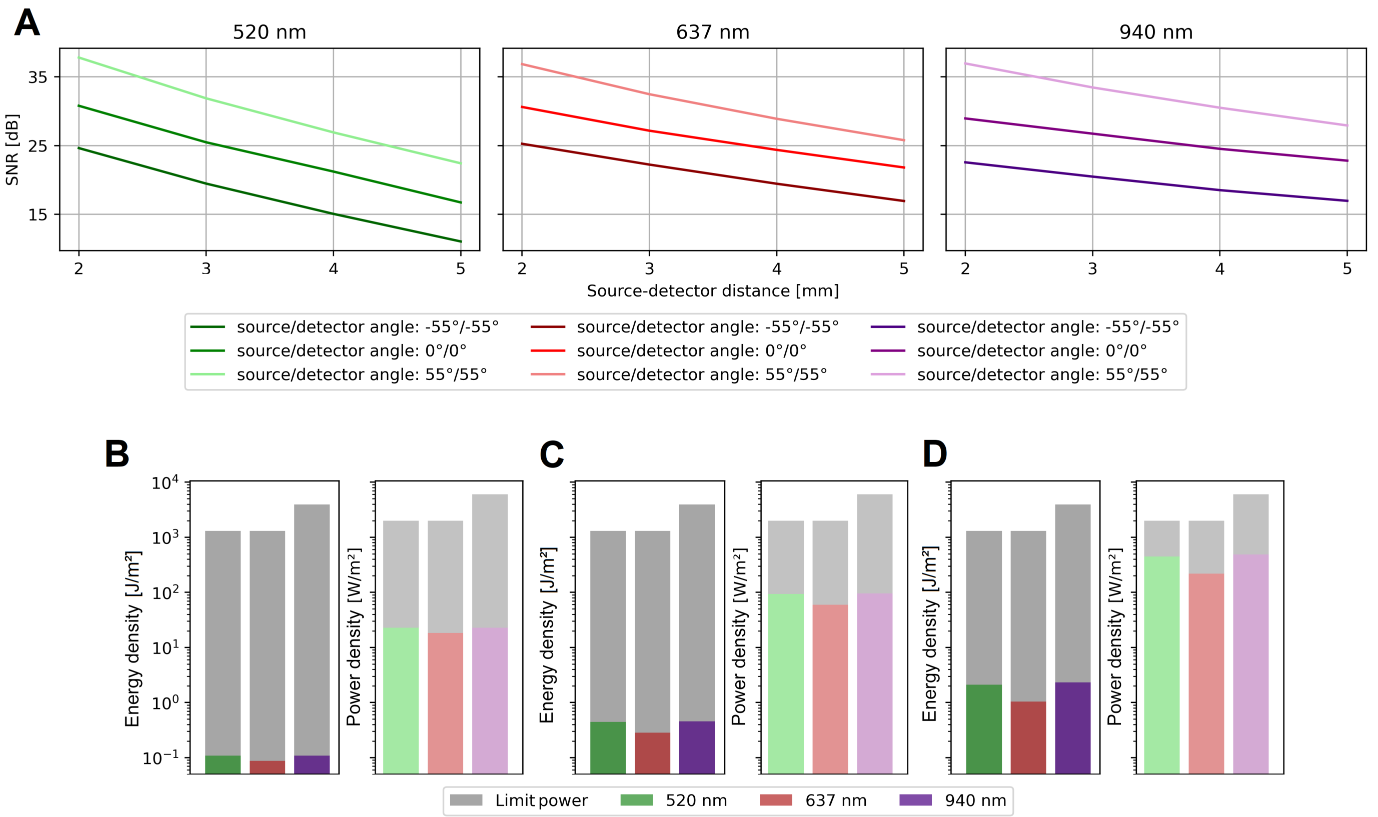

Fig. 3. A: SNR for source-detector angles of 55°, 0°, and −55°, all wavelengths and source-detector distances of 2 to 5 mm. B-D: MPE energy and power density limits, actual energy and power density. B: Energy and power density at source-detector angle of 55°. C: Energy and power density at source-detector angle of −55° to match SNR at 0°. D: Energy and power density at source-detector angle of −55° to match SNR at 55°.

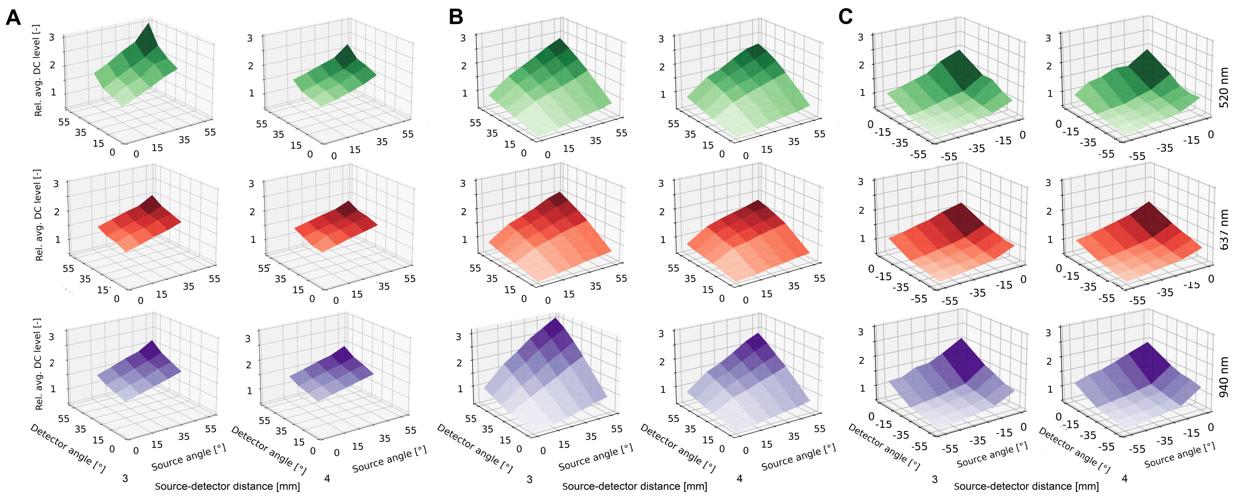

Fig. 4. Exemplary comparison of average DC level between laboratory measurements and MC simulation of HG across wavelengths, all measured angles, and source-detector distances d 3 and 4 mm. DC level was measured and simulated at the detector surface. Positive angle plots were normalised to the respective DC level at source angle of 15° and detector angle of 25° and negative plots at a source angle of −15° and detector angle of −25°. Wavelengths: 520 nm, 637 nm, 940 nm. A: laboratory measurements. B: MC simulations of HG of positive angles. C: MC simulations of HG of negative angles.

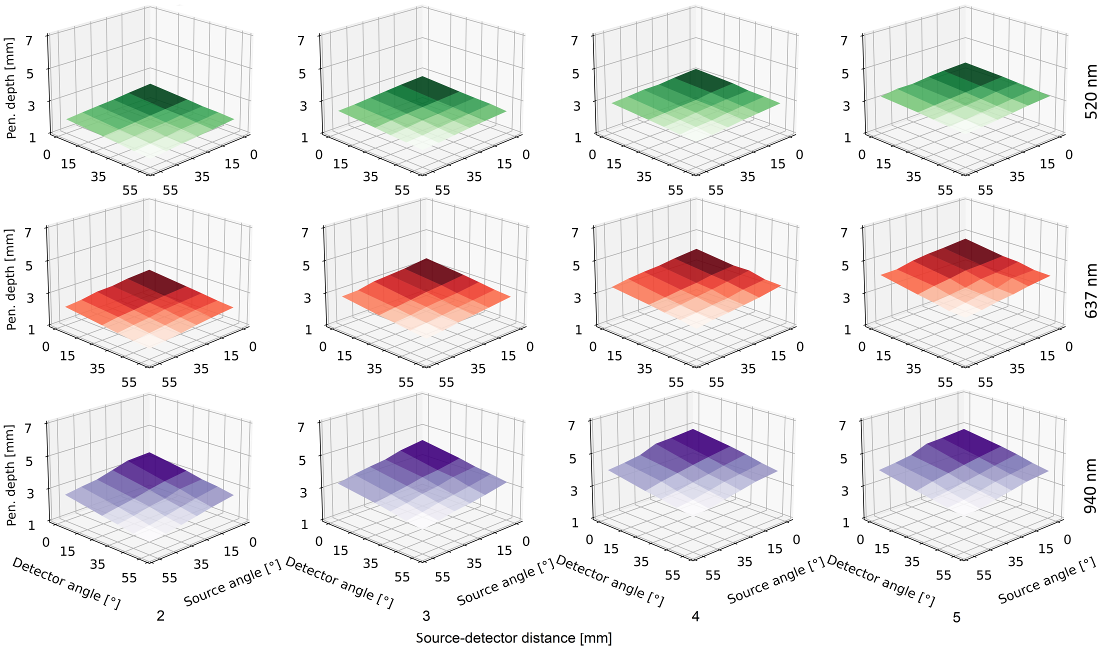

Fig. 5. Average maximum penetration depth of HG for source-detector distance d between 2 to 5 mm, plotted for each positive source and detector angle combination and all wavelength. 520 nm in green, 637 nm in red, 940 nm in purple.

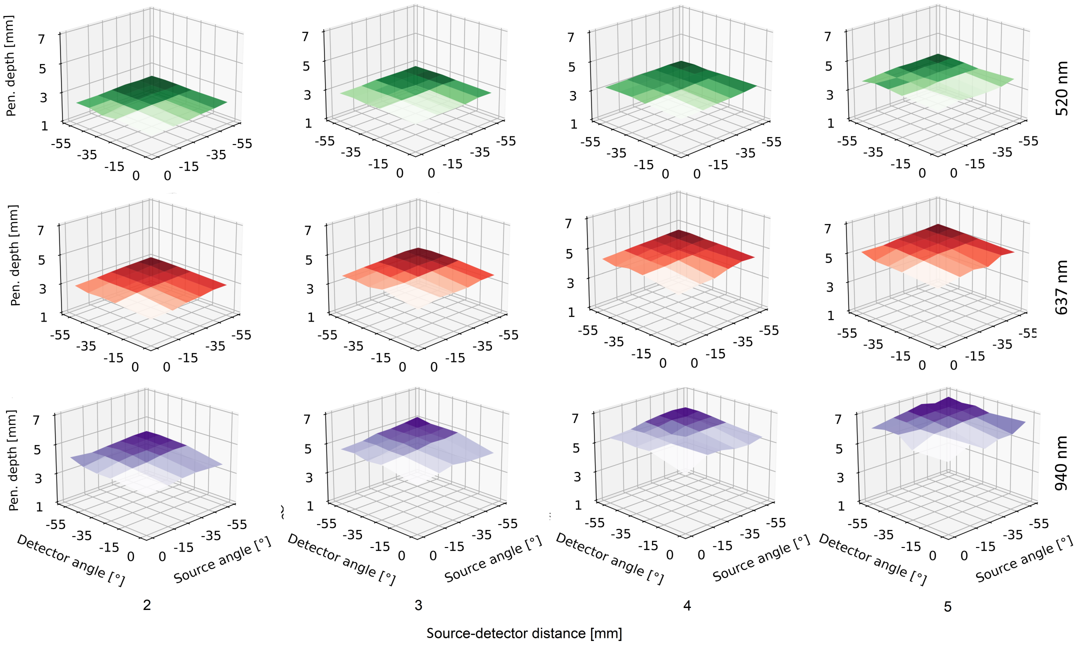

Fig. 6. Average maximum penetration depth of HG for source-detector distance d between 2 to 5 mm, plotted for each negative source and detector angle combination and all wavelength. 520 nm in green, 637 nm in red, 940 nm in purple.

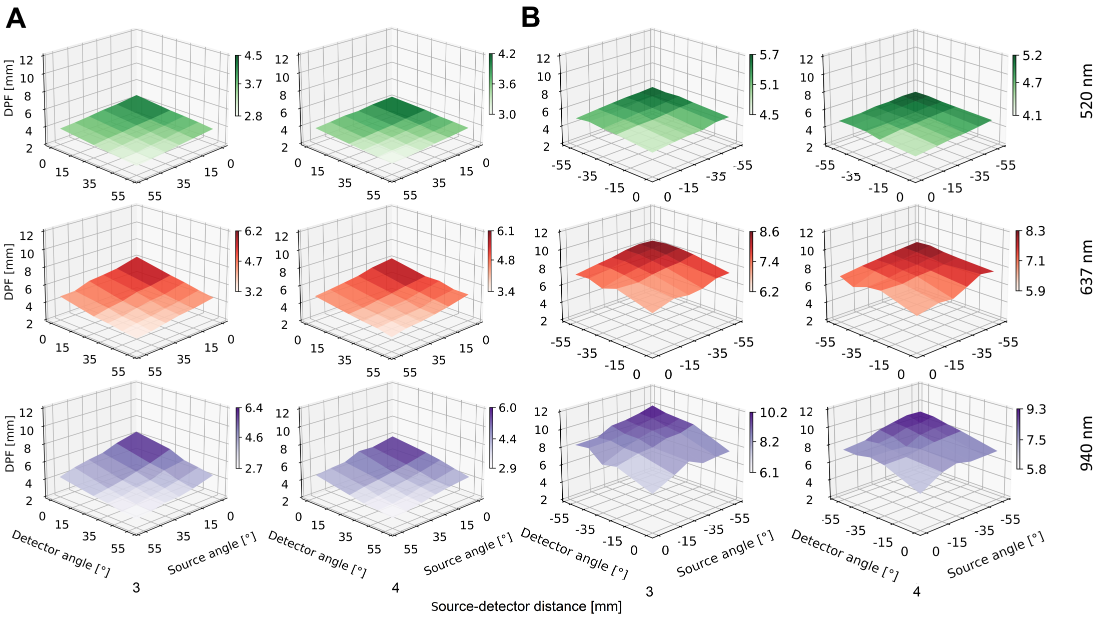

Fig. 7. Average differential pathlength factor (DPF) of HG for source-detector distance d of 3 and 4 mm, plotted for each positive and negative source and detector angle combination and all wavelength. 520 nm in green, 637 nm in red, 940 nm in purple. A: MC simulations of HG of positive angles. B: MC simulations of HG of negative angles.
